# Methyl 2-(4a,8-Dimethyl-7-oxodeca­hydro­naphthalen-2-yl)acrylate

**DOI:** 10.1107/S1600536812029303

**Published:** 2012-07-07

**Authors:** Mohamed Tebbaa, Ahmed Benharref, Jean-Claude Daran, Latifa Barkaoui, Moha Berraho

**Affiliations:** a Laboratoire de Chimie Bioorganique et Analytique, URAC 22, BP 146, FSTM, Université Hassan II, Mohammedia-Casablanca 20810 Mohammedia, Morocco; b Laboratoire de Chimie Biomoleculaire, Substances Naturelles et Réactivite, URAC16, Université Cadi Ayyad, Faculté des Sciences Semlalia, BP 2390, Bd My Abdellah, 40000 Marrakech, Morocco; c Laboratoire de Chimie de Coordination, 205 route de Narbonne, 31077 Toulouse Cedex 04, France

## Abstract

The title compound, C_16_H_24_O_3_, was isolated from the aerial part of *Inula Viscosa­* (*L*) Aiton [or *Dittrichia Viscosa­* (*L*) Greuter]. The mol­ecule contains two fused (*trans*) six-membered rings which both exibit a chair conformation. In the crystal, mol­ecules are linked into chains along [100] by weak C—H⋯O hydrogen bonds involving the methyl and carbonyl groups.

## Related literature

For the synthesis of the title compound, see: Barrero *et al.* (2009 ▶). For the medicinal inter­est in *Inula Viscosa­* (*L*) Aiton [or *Dittrichia Viscosa­* (*L*) Greuter], see: Shtacher & Kasshman (1970 ▶); Bohlmann *et al.* (1977 ▶); Chiappini *et al.* (1982 ▶). For the pharmacological inter­est, see: Azoulay *et al.* (1986 ▶); Bohlmann *et al.* (1977 ▶); Ceccherelli *et al.* (1988 ▶). For background to phytochemical studies of plants, see: Geissman & Toribio (1967 ▶). For conformational analysis, see: Cremer & Pople (1975 ▶).
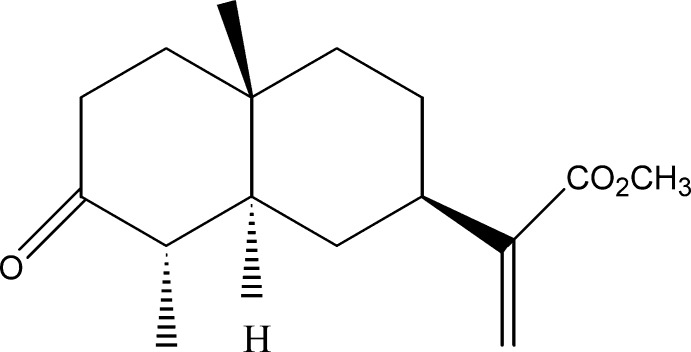


## Experimental

### 

#### Crystal data

C_16_H_24_O_3_
*M* *_r_* = 264.35
Tetragonal, 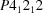

*a* = 7.3359 (1) Å
*c* = 54.7419 (13) Å
*V* = 2945.96 (9) Å^3^
*Z* = 8
Cu *K*α radiation
μ = 0.64 mm^−1^
*T* = 180 K
0.48 × 0.24 × 0.18 mm

#### Data collection

Agilent Xcalibur Eos Gemini ultra diffractometer
Absorption correction: multi-scan (*CrysAlis PRO*; Agilent, 2010 ▶) *T*_min_ = 0.737, *T*_max_ = 1.000
11562 measured reflections
2319 independent reflections
2286 reflections with *I* > 2σ(*I*)
*R*_int_ = 0.027
θ_max_ = 62.0°

#### Refinement

*R*[*F* ^2^ > 2σ(*F* ^2^)] = 0.042
*wR*(*F* ^2^) = 0.107
*S* = 1.22
2319 reflections
176 parameters
H-atom parameters constrained
Δρ_max_ = 0.15 e Å^−3^
Δρ_min_ = −0.13 e Å^−3^

### 

Data collection: *CrysAlis PRO* (Agilent, 2010 ▶); cell refinement: *CrysAlis PRO*; data reduction: *CrysAlis PRO*; program(s) used to solve structure: *SHELXS97* (Sheldrick, 2008 ▶); program(s) used to refine structure: *SHELXL97* (Sheldrick, 2008 ▶); molecular graphics: *ORTEP-3 for Windows* (Farrugia, 1997 ▶)and *PLATON* (Spek, 2009 ▶); software used to prepare material for publication: *WinGX* (Farrugia, 1999 ▶).

## Supplementary Material

Crystal structure: contains datablock(s) I, global. DOI: 10.1107/S1600536812029303/fj2573sup1.cif

Structure factors: contains datablock(s) I. DOI: 10.1107/S1600536812029303/fj2573Isup2.hkl

Supplementary material file. DOI: 10.1107/S1600536812029303/fj2573Isup3.cml

Additional supplementary materials:  crystallographic information; 3D view; checkCIF report

## Figures and Tables

**Table 1:** Hydrogen-bond geometry (Å, °)

*D*—H⋯*A*	*D*—H	H⋯*A*	*D*⋯*A*	*D*—H⋯*A*
C14—H14*B*⋯O2^i^	0.96	2.54	3.113 (3)	118

Symmetry code: (i) 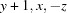.

**Fig. 1.**
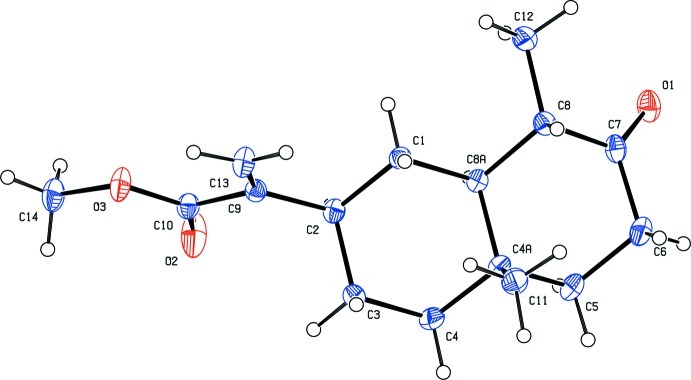
: Molecular structure of the title compound with the atom-labelling scheme. Displacement ellipsoids are drawn at the 30% probability. level. H atoms are represented as small spheres of arbitrary radii.

## supplementary crystallographic information

### Comment

The Inula Viscosa (*L*) is widespread in Mediterranean area and extends to the Atlantic cost of Morocco. It is a well known medicinal plant (Shtacher & Kasshman, 1970; Chiappini *et al.*, 1982) and has some pharmacological activities (Azoulay *et al.*, 1986). This plant has been the subject of chemical investigation in terms of isolating sesquiterpene lactones (Bohlmann *et al.*, 1977), sesquiterpene acids (Ceccherelli *et al.*, 1988; Geissman & Toribio, 1967). The ilicic acid is one of the main components of the dichloromethane extract of the Inula Viscosa (*L*) Aiton or Dittrichia Viscosa (*L*) Greuter]. In order to prepare products with high added value, that can be used in the pharmacologycal industry, we have studied the reactivity of this acid. Thus, from this acid, we have prepared by the method of Barrero *et al.* (2009), 2-(4a,8-Dimethyl-1, 2,3,4,4a,5,6,7- octahydro naphthalen-2-yl)-acrylic acid methyl ester. The epoxidation of the latter compound by metachloroperbenzoic acid (mCPBA), followed by the opening of the epoxide obtained by Bi(OTf)3 leads to the title compound (I) with a yield of 70%. The cristal structure of (I) is determined herin. The molecule is built up from two fused six-membered rings. The molecular structure of (I),Fig.1, shows the two rings to adopt a perfect chair conformation as indicated by Cremer & Pople (1975) puckering parameters Q(T)= 0.580 (2) Å and spherical polar angle θ = 180.0 (2)° with φ = 120 (9)° for the first ring (C1,C2··· C8A) and Q(T)= 0.572 (2) Å with a spherical polar angle θ = 175.9 (2)° and φ = 139 (3)° for the second ring (C4A, C5···C8A)(Cremer and Pople,1975). Molecules are linked by intermolecular C—H···O hydrogen bonds (Table 1) involving O2 and H14B atoms and propagating into three dimensional network.

### Experimental

To 2 g (8 mmol) of 2-(4a,8-Dimethyl-1,2,3,4,4a,5,6,7-octahydro-naphthalen-2-yl)- acrylic acid methyl ester dissolved in 50 ml of dichloromethane was added one equivalent of *m*-chloroperbenzoic acid at 70%. The reaction mixture was stirred at room temperature for 3 h, then treated three times with a solution of sodium bisulfite at 10%. The organic layer was then washed with distilled water three times until neutralization, dried over sodium sulfate, filtered and concentrated under reduced pressure. The residue obtained was chromatographed on silica gel eluting with hexane/ ethyl acetate (98/2) to give quantitatively the corresponding epoxide. 1 g (3.78 mmol) of this epoxyde is dissolved with 5% of boron trifluoride etherate (BF3.Et2O) in 20 ml of dichloromethane. The reaction mixture was left stirring for a period of half an hour and then treated with 20 ml of a solution of sodium bicarbonate to 10%. The organic layer was dried filtered and concentrated under reduced pressure. Chromatography on silica gel, eluting with hexane/ethyl acetate (98/2) of the residue obtained, allowed us to obtain 700 mg (2.64 mmol)of the title compound which was recrystallized in dichloromethane.

### Refinement

All H atoms were fixed geometrically and treated as riding with C—H = 0.93 Å(aromatic), 0.96 Å (methyl), 0.97 Å (methylene), 0.98 Å (methine) with *U*_iso_(H) = 1.2U_eq_ (aromatic, methylene, methine) or *U*_iso_(H) = 1.5*U*_eq_ (methyl). In the absence of significant anomalous scattering, the absolute configuration could not be reliably determined and any references to the Flack parameter were removed.

### Crystal data

**Table:**

C_16_H_24_O_3_	*D*_x_ = 1.192 Mg m^−^^3^
*M**_r_* = 264.35	Cu *K*α radiation, λ = 1.54184 Å
Tetragonal, *P*4_1_2_1_2	Cell parameters from 6272 reflections
Hall symbol: P 4abw 2nw	θ = 3.2–61.9°
*a* = 7.3359 (1) Å	µ = 0.64 mm^−^^1^
*c* = 54.7419 (13) Å	*T* = 180 K
*V* = 2945.96 (9) Å^3^	Box, colorless
*Z* = 8	0.48 × 0.24 × 0.18 mm
*F*(000) = 1152	

### Data collection

**Table:**

Agilent Xcalibur Eos Gemini ultra diffractometer	2319 independent reflections
Radiation source: Enhance Ultra (Cu) X-ray Source	2286 reflections with *I* > 2σ(*I*)
Miror monochromator	*R*_int_ = 0.027
Detector resolution: 16.1978 pixels mm^-1^	θ_max_ = 62.0°, θ_min_ = 3.2°
ω scan	*h* = −8→8
Absorption correction: multi-scan (*CrysAlis PRO*; Agilent, 2010)	*k* = −7→8
*T*_min_ = 0.737, *T*_max_ = 1.000	*l* = −61→62
11562 measured reflections	

### Refinement

**Table:**

Refinement on *F*^2^	Secondary atom site location: difference Fourier map
Least-squares matrix: full	Hydrogen site location: inferred from neighbouring sites
*R*[*F*^2^ > 2σ(*F*^2^)] = 0.042	H-atom parameters constrained
*wR*(*F*^2^) = 0.107	*w* = 1/[σ^2^(*F*_o_^2^) + (0.0378*P*)^2^ + 1.4663*P*] where *P* = (*F*_o_^2^ + 2*F*_c_^2^)/3
*S* = 1.22	(Δ/σ)_max_ < 0.001
2319 reflections	Δρ_max_ = 0.15 e Å^−^^3^
176 parameters	Δρ_min_ = −0.13 e Å^−^^3^
0 restraints	Extinction correction: *SHELXL97* (Sheldrick, 2008), Fc^*^=kFc[1+0.001xFc^2^λ^3^/sin(2θ)]^-1/4^
Primary atom site location: structure-invariant direct methods	Extinction coefficient: 0.0079 (4)

### Special details

**Table:**

Experimental. Empirical absorption correction using spherical harmonics, implemented in SCALE3 ABSPACK scaling algorithm. CrysAlisPro (Agilent Technologies, 2010)
Geometry. All s.u.'s (except the s.u. in the dihedral angle between two l.s. planes) are estimated using the full covariance matrix. The cell s.u.'s are taken into account individually in the estimation of s.u.'s in distances, angles and torsion angles; correlations between s.u.'s in cell parameters are only used when they are defined by crystal symmetry. An approximate (isotropic) treatment of cell s.u.'s is used for estimating s.u.'s involving l.s. planes.
Refinement. Refinement of *F*^2^ against ALL reflections. The weighted *R*-factor *wR* and goodness of fit *S* are based on *F*^2^, conventional *R*-factors *R* are based on *F*, with *F* set to zero for negative *F*^2^. The threshold expression of *F*^2^ > 2σ(*F*^2^) is used only for calculating *R*-factors(gt) *etc*. and is not relevant to the choice of reflections for refinement. *R*-factors based on *F*^2^ are statistically about twice as large as those based on *F*, and *R*- factors based on ALL data will be even larger.

### Fractional atomic coordinates and isotropic or equivalent isotropic displacement parameters (Å^2^)

**Table:**

	*x*	*y*	*z*	*U*_iso_*/*U*_eq_	
C1	1.0927 (3)	0.8703 (3)	0.07690 (4)	0.0245 (5)	
H1A	1.2083	0.8472	0.0849	0.029*	
H1B	1.0311	0.9671	0.0857	0.029*	
C2	1.1272 (3)	0.9311 (3)	0.05053 (4)	0.0261 (5)	
H2	1.1913	0.8313	0.0423	0.031*	
C3	0.9461 (3)	0.9583 (4)	0.03717 (4)	0.0342 (6)	
H3A	0.8808	1.0594	0.0445	0.041*	
H3B	0.9699	0.9887	0.0202	0.041*	
C4	0.8288 (3)	0.7880 (4)	0.03835 (4)	0.0368 (6)	
H4A	0.8897	0.6905	0.0296	0.044*	
H4B	0.7139	0.8118	0.0302	0.044*	
C4A	0.7903 (3)	0.7247 (3)	0.06465 (4)	0.0293 (5)	
C5	0.6931 (4)	0.5403 (4)	0.06370 (4)	0.0413 (6)	
H5A	0.7621	0.4583	0.0533	0.050*	
H5B	0.5739	0.5567	0.0564	0.050*	
C6	0.6696 (4)	0.4520 (4)	0.08907 (4)	0.0406 (6)	
H6A	0.5858	0.5240	0.0988	0.049*	
H6B	0.6188	0.3307	0.0873	0.049*	
C7	0.8502 (3)	0.4407 (3)	0.10176 (4)	0.0300 (5)	
C8	0.9541 (3)	0.6181 (3)	0.10379 (3)	0.0265 (5)	
H8	0.8789	0.7032	0.1132	0.032*	
C8A	0.9762 (3)	0.6979 (3)	0.07764 (4)	0.0234 (5)	
H8A	1.0423	0.6059	0.0682	0.028*	
C9	1.2453 (3)	1.0976 (3)	0.04836 (4)	0.0247 (5)	
C10	1.3641 (3)	1.1046 (3)	0.02623 (4)	0.0286 (5)	
C11	0.6688 (3)	0.8646 (4)	0.07742 (4)	0.0395 (6)	
H11A	0.5602	0.8834	0.0680	0.059*	
H11B	0.7334	0.9778	0.0790	0.059*	
H11C	0.6364	0.8205	0.0933	0.059*	
C12	1.1327 (3)	0.5938 (3)	0.11736 (4)	0.0354 (6)	
H12A	1.1111	0.5291	0.1323	0.053*	
H12B	1.1839	0.7112	0.1210	0.053*	
H12C	1.2161	0.5257	0.1074	0.053*	
C13	1.2460 (3)	1.2350 (3)	0.06410 (4)	0.0344 (6)	
H13A	1.3205	1.3355	0.0613	0.041*	
H13B	1.1721	1.2308	0.0779	0.041*	
C14	1.5954 (4)	1.2590 (4)	0.00496 (4)	0.0429 (7)	
H14A	1.6742	1.1545	0.0051	0.064*	
H14B	1.6673	1.3680	0.0062	0.064*	
H14C	1.5271	1.2611	−0.0100	0.064*	
O1	0.9105 (2)	0.2973 (2)	0.10919 (3)	0.0385 (4)	
O2	1.3651 (3)	0.9897 (3)	0.01066 (3)	0.0601 (6)	
O3	1.4716 (2)	1.2492 (2)	0.02541 (3)	0.0393 (5)	

### Atomic displacement parameters (Å^2^)

**Table:**

	*U*^11^	*U*^22^	*U*^33^	*U*^12^	*U*^13^	*U*^23^
C1	0.0237 (12)	0.0234 (12)	0.0265 (10)	−0.0016 (9)	−0.0015 (9)	−0.0006 (9)
C2	0.0271 (13)	0.0245 (12)	0.0267 (10)	−0.0025 (9)	0.0026 (9)	−0.0036 (9)
C3	0.0344 (14)	0.0428 (15)	0.0254 (10)	−0.0081 (11)	−0.0019 (10)	0.0071 (10)
C4	0.0315 (14)	0.0503 (16)	0.0287 (11)	−0.0116 (12)	−0.0069 (10)	0.0017 (11)
C4A	0.0221 (12)	0.0352 (13)	0.0307 (11)	−0.0037 (10)	−0.0012 (9)	0.0018 (10)
C5	0.0332 (15)	0.0448 (16)	0.0459 (14)	−0.0175 (12)	−0.0064 (11)	0.0034 (12)
C6	0.0326 (15)	0.0385 (15)	0.0507 (14)	−0.0120 (12)	−0.0002 (11)	0.0078 (12)
C7	0.0321 (13)	0.0284 (13)	0.0297 (10)	−0.0008 (11)	0.0103 (9)	0.0010 (10)
C8	0.0272 (12)	0.0267 (12)	0.0256 (10)	0.0021 (10)	0.0031 (9)	0.0001 (9)
C8A	0.0225 (11)	0.0218 (12)	0.0257 (10)	0.0006 (9)	0.0022 (8)	−0.0025 (8)
C9	0.0242 (12)	0.0223 (12)	0.0275 (10)	0.0004 (10)	0.0007 (8)	0.0010 (9)
C10	0.0312 (13)	0.0230 (12)	0.0316 (11)	−0.0036 (10)	0.0012 (9)	−0.0016 (9)
C11	0.0239 (13)	0.0462 (16)	0.0483 (14)	0.0076 (12)	0.0026 (11)	0.0121 (12)
C12	0.0369 (14)	0.0326 (14)	0.0367 (12)	−0.0039 (11)	−0.0062 (10)	0.0078 (10)
C13	0.0333 (13)	0.0310 (13)	0.0389 (12)	−0.0066 (11)	0.0098 (10)	−0.0038 (11)
C14	0.0352 (14)	0.0504 (17)	0.0430 (14)	−0.0076 (13)	0.0161 (11)	0.0028 (12)
O1	0.0422 (11)	0.0254 (10)	0.0479 (9)	−0.0015 (8)	0.0084 (8)	0.0057 (7)
O2	0.0819 (16)	0.0494 (12)	0.0490 (10)	−0.0310 (11)	0.0323 (10)	−0.0215 (10)
O3	0.0392 (10)	0.0375 (10)	0.0411 (9)	−0.0152 (8)	0.0162 (7)	−0.0061 (7)

### Geometric parameters (Å, º)

**Table:**

C1—C8A	1.527 (3)	C7—O1	1.211 (3)
C1—C2	1.532 (3)	C7—C8	1.512 (3)
C1—H1A	0.9700	C8—C12	1.517 (3)
C1—H1B	0.9700	C8—C8A	1.555 (3)
C2—C9	1.502 (3)	C8—H8	0.9800
C2—C3	1.529 (3)	C8A—H8A	0.9800
C2—H2	0.9800	C9—C13	1.326 (3)
C3—C4	1.519 (3)	C9—C10	1.493 (3)
C3—H3A	0.9700	C10—O2	1.199 (3)
C3—H3B	0.9700	C10—O3	1.322 (3)
C4—C4A	1.539 (3)	C11—H11A	0.9600
C4—H4A	0.9700	C11—H11B	0.9600
C4—H4B	0.9700	C11—H11C	0.9600
C4A—C11	1.529 (3)	C12—H12A	0.9600
C4A—C5	1.530 (3)	C12—H12B	0.9600
C4A—C8A	1.551 (3)	C12—H12C	0.9600
C5—C6	1.542 (3)	C13—H13A	0.9300
C5—H5A	0.9700	C13—H13B	0.9300
C5—H5B	0.9700	C14—O3	1.443 (3)
C6—C7	1.498 (3)	C14—H14A	0.9600
C6—H6A	0.9700	C14—H14B	0.9600
C6—H6B	0.9700	C14—H14C	0.9600
			
C8A—C1—C2	111.03 (17)	O1—C7—C8	122.6 (2)
C8A—C1—H1A	109.4	C6—C7—C8	115.6 (2)
C2—C1—H1A	109.4	C7—C8—C12	111.72 (19)
C8A—C1—H1B	109.4	C7—C8—C8A	107.99 (17)
C2—C1—H1B	109.4	C12—C8—C8A	113.89 (18)
H1A—C1—H1B	108.0	C7—C8—H8	107.7
C9—C2—C3	110.93 (19)	C12—C8—H8	107.7
C9—C2—C1	114.00 (17)	C8A—C8—H8	107.7
C3—C2—C1	110.20 (18)	C1—C8A—C4A	112.01 (17)
C9—C2—H2	107.1	C1—C8A—C8	113.25 (17)
C3—C2—H2	107.1	C4A—C8A—C8	112.22 (17)
C1—C2—H2	107.1	C1—C8A—H8A	106.2
C4—C3—C2	111.4 (2)	C4A—C8A—H8A	106.2
C4—C3—H3A	109.4	C8—C8A—H8A	106.2
C2—C3—H3A	109.4	C13—C9—C10	119.9 (2)
C4—C3—H3B	109.4	C13—C9—C2	124.7 (2)
C2—C3—H3B	109.4	C10—C9—C2	115.42 (18)
H3A—C3—H3B	108.0	O2—C10—O3	122.4 (2)
C3—C4—C4A	113.09 (18)	O2—C10—C9	123.8 (2)
C3—C4—H4A	109.0	O3—C10—C9	113.80 (18)
C4A—C4—H4A	109.0	C4A—C11—H11A	109.5
C3—C4—H4B	109.0	C4A—C11—H11B	109.5
C4A—C4—H4B	109.0	H11A—C11—H11B	109.5
H4A—C4—H4B	107.8	C4A—C11—H11C	109.5
C11—C4A—C5	109.7 (2)	H11A—C11—H11C	109.5
C11—C4A—C4	109.4 (2)	H11B—C11—H11C	109.5
C5—C4A—C4	108.70 (19)	C8—C12—H12A	109.5
C11—C4A—C8A	112.85 (18)	C8—C12—H12B	109.5
C5—C4A—C8A	108.26 (19)	H12A—C12—H12B	109.5
C4—C4A—C8A	107.80 (18)	C8—C12—H12C	109.5
C4A—C5—C6	113.1 (2)	H12A—C12—H12C	109.5
C4A—C5—H5A	109.0	H12B—C12—H12C	109.5
C6—C5—H5A	109.0	C9—C13—H13A	120.0
C4A—C5—H5B	109.0	C9—C13—H13B	120.0
C6—C5—H5B	109.0	H13A—C13—H13B	120.0
H5A—C5—H5B	107.8	O3—C14—H14A	109.5
C7—C6—C5	110.0 (2)	O3—C14—H14B	109.5
C7—C6—H6A	109.7	H14A—C14—H14B	109.5
C5—C6—H6A	109.7	O3—C14—H14C	109.5
C7—C6—H6B	109.7	H14A—C14—H14C	109.5
C5—C6—H6B	109.7	H14B—C14—H14C	109.5
H6A—C6—H6B	108.2	C10—O3—C14	116.22 (19)
O1—C7—C6	121.8 (2)		

### Hydrogen-bond geometry (Å, º)

**Table:**

*D*—H···*A*	*D*—H	H···*A*	*D*···*A*	*D*—H···*A*
C14—H14*B*···O2^i^	0.96	2.54	3.113 (3)	118

Symmetry code: (i) *y*+1, *x*, −*z*.
